# Therapeutic Chemical Screen Identifies Phosphatase Inhibitors to Reconstitute PKB Phosphorylation and Cardiac Contractility in ILK-Deficient Zebrafish

**DOI:** 10.3390/biom8040153

**Published:** 2018-11-19

**Authors:** Alexander Pott, Maryam Shahid, Doreen Köhler, Christian Pylatiuk, Karolina Weinmann, Steffen Just, Wolfgang Rottbauer

**Affiliations:** 1 Department of Internal Medicine II, Ulm University, Albert-Einstein-Allee 23, D-89081 Ulm, Germany; alexander_pott@web.de (A.P.); maya873@gmail.com (M.S.); karolina.weinmann@uniklinik-ulm.de (K.W.); 2 Department of Internal Medicine III, University of Heidelberg, D-69120 Heidelberg, Germany; doreen.koehler@med.uni-heidelberg.de; 3 Institute of Applied Computer Science, Karlsruhe Institute of Technology, D-76344 Eggenstein-Leopoldshafen, Germany; pylatiuk@kit.edu

**Keywords:** dilated cardiomyopathy, integrin-linked kinase-protein kinase B (ILK-PKB) signaling, small chemical compounds, phosphatase inhibitors

## Abstract

Patients with inherited dilated cardiomyopathy (DCM) often suffer from severe heart failure based on impaired cardiac contractility leading to increased morbidity and mortality. Integrin-linked kinase (ILK) as a part of the cardiac mechanical stretch sensor was found to be an essential genetic regulator of cardiac contractility. Integrin-linked kinase localizes to z-disks and costameres in vertebrate hearts and regulates the activity of the signaling molecule protein kinase B (PKB/Akt) by controlling its phosphorylation. Despite identification of several potential drug targets in the ILK signaling pathway, pharmacological treatment strategies to restore contractile function in ILK-dependent cardiomyopathies have not been established yet. In recent years, the zebrafish has emerged as a valuable experimental system to model human cardiomyopathies as well as a powerful tool for the straightforward high-throughput in vivo small compound screening of therapeutically active substances. Using the ILK deficient zebrafish heart failure mutant *main squeeze* (*msq*), which shows reduced PKB phosphorylation and thereby impaired cardiac contractile force, we identified here, in an automated small compound screen, the protein phosphatase inhibitors calyculin A and okadaic acid significantly restoring myocardial contractile function by reconstituting PKB phosphorylation in *msq* ILK-deficient zebrafish embryos.

## 1. Introduction

Dilated cardiomyopathy (DCM) is a life-threatening heart disease significantly contributing to systolic heart failure and sudden cardiac death based on reduced cardiac contractility [1,2,3,4]. Molecular and genetic studies have identified more than 30 different DCM disease genes, mainly coding for proteins of the sarcomere, the cardiac Z-disc and the cytoskeleton [5].

To allow adaption of cardiac contractility on changing circulatory demands such as arterial blood pressure or volume preload, the autoregulatory cardiac stretch sensor system translates biomechanical strain of cardiomyocytes into activation of several signaling pathways regulating myocardial contractile force in vertebrates. Congruously, mutations of genes coding for proteins of the cardiac mechanical stress sensor are known to cause DCM in humans [6,7]. However, the detailed genetic and molecular underpinnings of this complex autoregulatory mechanism are not fully understood yet, but of high clinical importance, since impaired adaption of cardiac contractility is considered to cause a sizeable proportion of DCM-related heart failure cases in humans [2,6,8]. Genetic studies of cardiac stretch sensor components in zebrafish, mice and humans identified the integrin-linked kinase (ILK) as an essential regulator of cardiac contractility adaption on changing circulatory demands [7,9].

**Figure 1:**
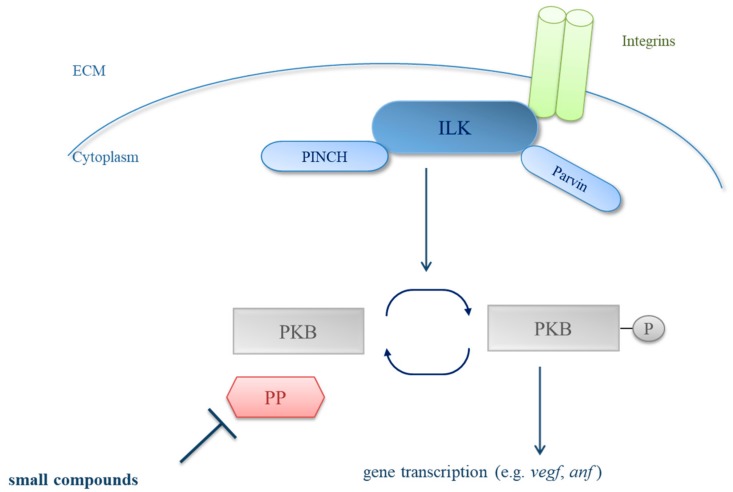
Schematic illustration of the integrin-linked kinase-protein kinase B (ILK-PKB) signaling pathway. Integrin-linked kinase forms, together with PINCH (particularly interesting Cys-His-rich protein) and parvin, the ILK-PINCH-parvin (IPP) complex and mediates signals from the extracellular matrix (ECM) to the cytoplasm through integrins. The phosphorylated downstream target PKB facilitates the expression of stretch responsive genes such as the *atrial natriuretic factor* (*anf*), thereby effectively transducing signals from the cardiac stretch sensor. Reduced PKB phosphorylation in ILK deficient *main squeeze* mutant zebrafish hearts was demonstrated to lead to impaired cardiac contractility and heart failure [10]. In this context, the inhibition of protein phosphatases (PP) by small chemical compounds that results in an increase of PKB phosphorylation might be a promising therapeutic approach to treat ILK-associated cardiomyopathies.

In a forward genetic screen, we identified the zebrafish DCM mutant *main squeeze* (*msq*), carrying a mutation in the kinase domain of ILK (L308P), leading to reduced kinase activity and finally to a loss of cardiac stretch sensor function. Accordingly, homozygous *msq* mutant embryos are characterized by severely reduced ventricular pump function as well as by decreased expression levels of stretch responsive genes such as the *atrial natriuretic factor* (*anf*) and *vascular endothelial growth factor* (*vegf*) [10]. Together with PINCH (particularly interesting Cys-His-rich protein) and β-parvin, ILK forms the functional ILK-PINCH-parvin (IPP) complex (Figure 1) [11,12], which is a crucial element of the cardiac stretch sensor [13,14]. Similar to the ILK-deficient *msq* mutant, ablation of β-parvin or PINCH in wild-type zebrafish leads to severely reduced cardiac contractility emphasizing that ILK as well as its interactors are essential regulators of ventricular pump function [15].

In vertebrates, ILK is mainly expressed in heart and skeletal muscle, where it interacts through integrins with growth factor receptors and signaling molecules such as the protein kinase B (PKB) for signal transduction from the extracellular matrix to the cytoplasm [16,17,18,19,20] (Figure 1). In line with this, PKB phosphorylation as a downstream target of ILK is severely reduced in *msq* zebrafish. Remarkably, overexpression of constitutive active PKB restores cardiac contractility of *msq* [10], indicating that PKB phosphorylation is critical for regular heart function. However, efficient pharmacological approaches to enhance PKB phosphorylation and activation have not been established yet, but might be crucial to improve contractile performance in vivo.

In recent years, the zebrafish has emerged as a powerful tool for high-throughput in vivo screening of small chemical compounds allowing biomolecule evaluation with straightforward assessment of essential cardiac parameters such as cardiac development, myocardial contractility and heart rhythm [21,22,23]. Using the zebrafish as drug screening platform, we aimed to identify chemical compounds rescuing heart failure in *msq* mutant embryos via maintenance of PKB phosphorylation. Hence, by using our automated small compound screening platform, we identified two phosphatase inhibitors, okadaic acid and calyculin A, to significantly improve ventricular pump function by enhancing PKB phosphorylation in ILK-deficient *msq* mutant zebrafish embryos.

## 2. Material and Methods

### 2.1. Zebrafish Strains

Zebrafish care and breeding was performed as described before [24]. All procedures and experiments in this study were carried out after appropriate institutional approvals (Tierforschungszentrum (TFZ) Ulm University, No. 0183), which conform to the EU Directive 2010/63/EU. For all procedures, the zebrafish strain *main squeeze*, *msq* (M347), was used [10].

### 2.2. Genotyping, Western Blot Analysis, and RNA In Situ Hybridization

Genotyping of *msq* embryos was performed by polymerase chain reaction (PCR) analysis using the satellite markers z7028 (fwd CAACACCAGCATAGCCATGT, rev TGTGACAAGGTCAGTGGAGC) as well as z7504 (fwd AATTGGGCTGCGTTTCATAC, rev TTCCACCTCCTGTAACCTGC) after DNA isolation of whole embryos. Protein extraction for Western blot analysis was performed from whole zebrafish embryos. For immunoblotting the proteins were separated by sodium dodecyl sulfate-polyacrylamide gel electrophoresis (SDS-PAGE) and transferred to a polyvinylidene fluoride (PVDF) membrane. The blots were probed with the primary antibody anti-pPKB S347 (4058, NEB/Cell Signaling, Danvers, MA, USA). Anti-pan-Cadherin (ab16505, Abcam, Cambridge, MA, USA) served as loading control. Signals were detected by chemiluminescence (anti-rabbit-HRP). Ribonucleic acid whole-mount in situ hybridization was used to detect expression of *anf* transcripts essentially as described elsewhere [10].

### 2.3. Small Compound Screen and Functional Assessment in Main Squeeze Embryos

Small compound screening was performed using a modified phosphatase inhibitor library with a total of 32 different small molecules (BML-2834, ENZO Life Sciences, Inc., Farmingdale, NY, USA and BIOZOL GmbH, Eching, Germany, Table A1 in the Appendix A). At 48 h post fertilization (hpf) stage-matched wild-type siblings and *msq* mutant embryos (divided based on the heart failure phenotype) were individually transferred into a 96-well-plate and ten embryos (five mutants and five siblings) tested and analyzed per compound using our established automated small compound screening platform [21]. Small compounds were added with a final concentration of 10 µM except for the compounds A1–A3. To avoid toxic side-effects of the protein phosphatase (PP1 and PP2A) inhibitors, which are associated with tumor promotion as well as impaired liver and gastrointestinal function in animals as well as humans, we applied concentrations for calyculin A (A1), cyclosporine A (A2) and okadiac Acid (A3) referring to previous in vivo studies (A1: 0.1 µM, A2: 0.15 µM, A3: 0.75 µM) [25,26,27]. Dimethyl sulfoxide (DMSO) was used as a solvent control with a concentration of 0.1%. Embryos were treated and incubated for 24 h and kept in an incubator at 29 °C. Since proper cardiac development requires regular myocardial contractions, we expected an additional developmental rescue effect in case of early drug treatment [28]. Hence, incubation period was extended from 4 to 96 hpf in the secondary drug screening for the compounds calyculin A, okadaic acid and cyclosporine A. Impact of small molecules on cardiac contractile function was evaluated by video microscopic movies of zebrafish hearts with the DM IRB (Leica, Wetzlar, Germany) microscope. The functional assessment of cardiac contractility was carried out as described before [24,29]. Fractional shortening (FS) and ventricular diameters were measured with the help of the zebraFS software (http://www.benegfx.de). Only data from experiments where fractional shortening for at least three embryos could be measured, were included in statistical analysis.

### 2.4. Statistical Analysis

If not further specified, results are expressed as mean and standard deviation (mean ± S.D.). Significance of differences of numeric values between two groups was calculated by t-test if normal distribution with equal variance was given. Normal distribution was determined by Shapiro–Wilk test and equal variance by Brown–Forsythe test. Numeric variables that were not normally distributed were analyzed by Mann–Whitney rank sum test. A *p*-value <0.05 was considered significant. In case of multiple testing per data-set *p*-value was adapted by the Bonferroni adjustment method. Statistical assessment was performed with Excel (Version 2016, Microsoft Inc., Redmond, WA, USA) or XLStat software (V 2016.02.28430, Addinsoft, New York, NY, USA).

## 3. Results

### 3.1. Primary Small Compound Screen Identifies Phosphatase Inhibitors Restoring Cardiac Contractility in msq Zebrafish

By genetically and molecularly characterizing the zebrafish mutant *main squeeze* (*msq*), we identified ILK to be crucial to guarantee cardiac contractile force by controlling PKB phosphorylation and thereby its activity [10,15].

Since PKB activity is significantly reduced in *msq* embryos, we here performed drug screening of a phosphatase inhibitor library containing 32 different compounds (Table A1 in the Appendix A) solved in DMSO using our established automated screening platform [21] with the aim to restore PKB phosphorylation and subsequently systolic ventricular pump function.

**Figure 2:**
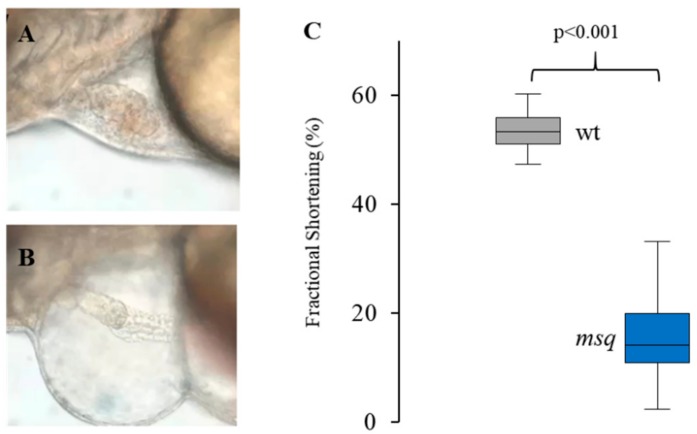
*msq* mutants show cardiac edema and reduced contractile function. *msq* mutants can be distinguished from their wild-type siblings at 48 h post fertilization (hpf). (**A**–**C**) Starting from this developmental stage, *msq* show cardiac edema (**B**) compared to wild-type sibling (**A**) and significantly decreased ventricular fractional shortening compared to wild-type controls at 72 hpf (**C**).

First, to study the impact of the solvent agent DMSO on cardiac contractility, we analyzed fractional shortening in wild-type (wt) and *msq* zebrafish incubated with 0.1% DMSO. We found that mean ventricular fractional shortening (FS) in wt treated with 0.1% DMSO was 53.2 ± 3.4% (*n* = 51) compared to 15.3 ± 6.7% (*n* = 35; *p* < 0.001) in *msq* with DMSO (Figure 2C). These findings are in line with previous reported data on ventricular FS in wt and *msq* zebrafish [10], indicating that cardiac contractility is affected in neither wt nor *msq* zebrafish by the solvent agent DMSO.

During small compound screening, as many as 8/32 (25.0%) compounds in wild-type zebrafish and 8/32 (25.0%) compounds in *msq* mutants led to lethality of more than 50% of compound treated zebrafish embryos. Except for the tyrosine phosphatase inhibitor *RK-682* (B6) that was lethal in *msq*, and except for Deltamethrin (B12) that was lethal in wt zebrafish, all compounds leading to death in wild-type zebrafish were also lethal in *msq* (Table A1). In both study groups, *Endothall* (B3) was excluded from further statistical analysis, since more than 50% of zebrafish in the DMSO control group died. Thus, fractional shortening data were available for 23 (71.9%) compounds applied in both groups, wild-type siblings and mutant *msq* zebrafish embryos.

**Figure 3:**
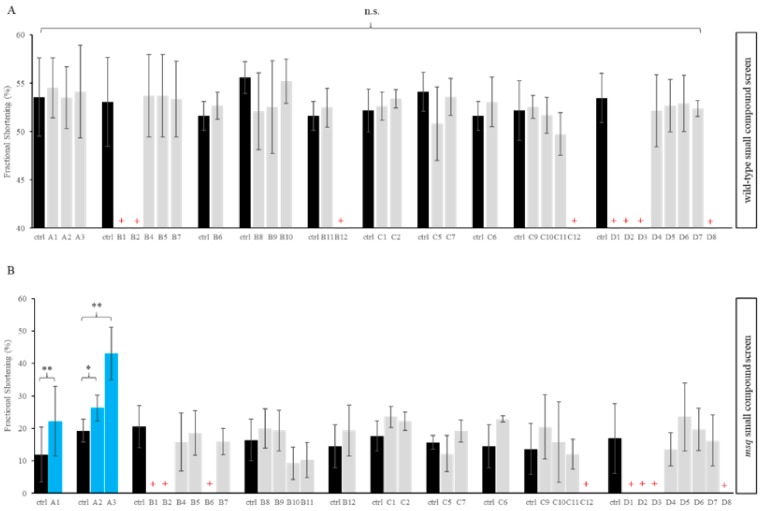
Primary small compound screen in *msq* mutants revealed three compounds to reconstitute fractional shortening (FS). Impact of tested compounds on ventricular fractional shortening in wild-type siblings as well as *msq* mutants. The data for average fractional shortening are plotted only for the compounds where at least three embryos could be quantified. (**A**) None of the embryos showed any increase or decrease in the average fractional shortening in wild-type embryos, whereas (**B**) compound A1, A2 and A3 results in significantly increased ventricular FS in *msq* mutants (*: Statistically significant, **: Highly significant, **+**: Lethal). For the drugs’ names, please refer to the Table A1 in the Appendix A.

As shown in Figure 3A, none of the applied small compounds led to a significant increase of ventricular FS in wild-type siblings compared to the DMSO control group (Figure 3A). By contrast, 3 out of 32 (9.4%) tested chemicals, namely A1 (calyculin A), A2 (cyclosporin A) and A3 (okadaic acid) showed a significant improvement of ventricular FS in *msq* mutants compared to *msq* controls treated with DMSO only at 72 hpf (Figure 3B). All other tested compounds did not show significant changes in FS in *msq* compared to DMSO treated mutants.

For *msq* mutants incubated with calyculin A, mean ventricular fractional shortening 24 h after drug administration was 22.2 ± 10.7% compared to 12.0 ± 8.5% in the control group (*p* = 0.006). Similarly, mean ventricular FS in *msq* mutants treated with Cyclosporine A was significantly higher (26.3 ± 4.0%) compared to the control group (19.3 ± 3.5%; *p* = 0.023). For okadaic acid, we found that mean ventricular FS in *msq* mutants was 43.0 ± 8.1% at 24 h after drug administration in comparison to a mean ventricular FS of 19.3 ± 3.5% in controls (*p* < 0.001; Figure 3B). Next, we performed a detailed investigation (secondary screen) of the three identified compounds, with the aim to get a more in-depth understanding of the molecular underpinnings leading to significant increase of ventricular FS in *msq* mutants.

### 3.2. Calyculin A and Okadaic Acid Reconstitute Cardiac Contractility in msq Cardiomyopathy via Restored PKB Phosphorylation

In order to confirm the findings of the primary drug screening and to evaluate the impact of the three identified compounds on cardiac contractility and PKB phosphorylation in more detail, we analyzed ventricular FS in wild-type siblings and *msq* mutants that were incubated with the candidate compounds from 4 to 96 hpf compared to 48 to 72 hpf in the initial drug screening.

**Figure 4:**
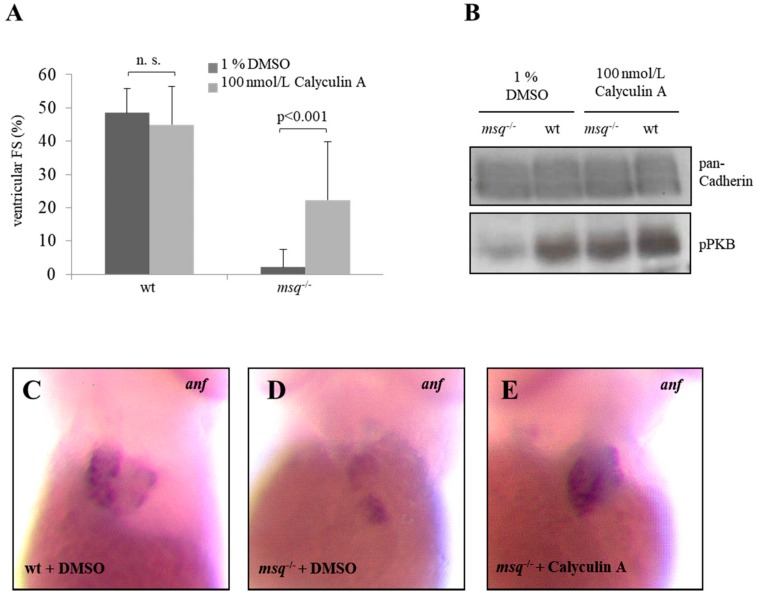
Calyculin A reconstitutes contractile force, PKB phosphorylation and *anf* expression in *msq*. (**A**) Ventricular FS of homozygous mutant *msq* embryos (*msq*^−/−^) in comparison to wild-type (WT) zebrafish. Homozygous *msq* embryos treated with 100 nmol/L calyculin A during 4–96 hpf displayed improved contractile force (25.4 ± 18.9% (*p* = 0.00015) compared to embryos incubated with DMSO (2.2 ± 5.3%). (n.s.: Not significant) (**B**) Treatment of homozygous *msq*^−/−^ embryos with 100 nmol/L calyculin A displayed an increased PKB phosphorylation (pPKB) in comparison to controls. In situ hybridization revealed that calyculin A treated homozygous *msq*^−/−^ embryos (**E**) show, in contrast to DMSO-treated wild-type zebrafish (**C**) and DMSO-treated *msq*^−/−^ embryos (**D**), an almost indistinguishable *anf* expression.

After 96 hpf, wild-type zebrafish treated with calyculin A present with ventricular FS of 43.6 ± 13.6% compared to 42.6 ± 12.7% in untreated wt embryos, indicating that calyculin A has no adverse effects on cardiac contractility in the wild-type situation. Remarkably, the nearly abolished cardiac contractility observed in untreated *msq* embryos (ventricular FS: 2.2 ± 5.3%) at 96 hpf was significantly improved in *msq* mutants (25.4 ± 18.9%; *p* < 0.001) treated with calyculin A after extended incubation time (Figure 4A). These data confirm the positive impact of calyculin A on ventricular FS in *msq* mutants and furthermore suggests that earlier drug administration and longer drug treatment leads to stable reconstitution of cardiac contractility also at 96 hpf.

Next, we evaluated the molecular impact of calyculin A, which is known to inhibit protein phosphatases such as PP1 and PP2A. For both, PP1 and PP2A, it has been shown that PKB is a physiological target in different tissues leading to decreased PKB phosphorylation [25,30]. To examine whether calyculin A improves cardiac contractility in *msq* zebrafish mutants via the reconstitution of PKB phosphorylation, we performed immunoblotting assays to assess the PKB phosphorylation status in *msq* mutants treated with calyculin A and their respective controls. Interestingly, we found that untreated *msq* zebrafish present with a weak PKB phosphorylation (pPKB) signal, whereas calyculin A treated *msq* embryos displayed high pPKB levels comparable to wild-type zebrafish (Figure 4B). These findings demonstrate that calyculin A treatment of ILK-deficient *msq* embryos effectively inhibits further PKB dephosphorylation, thereby reconstituting phospho-PKB levels and rescuing cardiac contractility in *msq* heart failure mutants.

To further reveal the effect of enhanced PKB activation by calyculin A on *anf* expression, which is a final downstream target of ILK-PKB signaling, we performed *anf* specific whole-mount antisense RNA in situ hybridizations. By in situ hybridization of individual *msq* embryos, we found that treatment with calyculin A results in a significantly increased *anf* expression similar to the situation in wild-type zebrafish (Figure 4C–E), further substantiating that calyculin A treatment is able to rescue heart failure of *msq* mutants on a functional but also molecular level.

Similar to calyculin A, the fatty acid okadaic acid is also known to act as inhibitor of the protein phosphatases PP1 and PP2A [30], insinuating that PKB phosphorylation might also be influenced by this compound.

**Figure 5:**
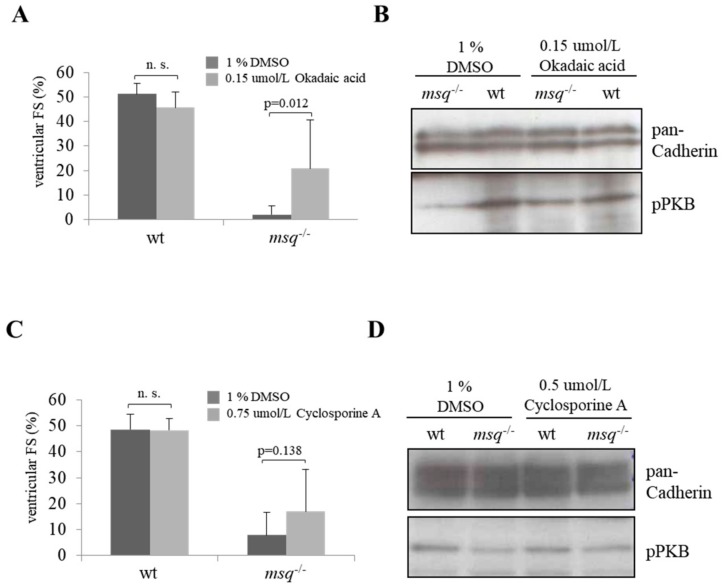
Okadaic acid and cyclosporine A partially reconstitute the contractile force and PKB phosphorylation in *msq*. (**A**) After treatment with 0.15 µmol/L okadaic acid during 4–96 hpf homozygous *msq*^−/−^ embryos showed an increased contractility from 2.0 ± 3.5% to 20.8 ± 19.9%. (**B**) Immunoblotting indicated that the phosphorylation status of PKB (pPKB) was also slightly increased in 0.15 µmol/L okadaic acid treated *msq*^−/−^ embryos (incubation during 4–96 hpf). (**C**) Ventricular fractional shortening of *msq*^−/−^ embryos treated with 0.75 µmol/L cyclosporine A during 4–96 hpf showed in comparison to DMSO-treated *msq*^−/−^ embryos (7.9 ± 8.7%) a partially reconstituted contractile force (16.9 ± 16.2%). (**D**) Correspondingly to fractional shortening, immunoblotting revealed that treatment with 0.5 µmol/L cyclosporine A (incubation during 48–72 hpf) slightly increased the level of phosphorylated PKB in *msq*^−/−^ embryos compared to controls. (n.s.: Not significant).

According to the extended calyculin A screening, we performed incubation of okadaic acid in *msq* embryos from 4 to 96 hpf. Wild-type zebrafish treated with okadaic acid present with a mean ventricular FS of 45.8 ± 8.2% and were indistinguishable from untreated wild-type embryos (ventricular FS: 51.2 ± 4.4%; *p* >0.05) four days post compound administration (Figure 5A). As expected, *msq* embryos of the DMSO-treated control group exhibited a mean ventricular FS of 2.0 ± 3.5%. In contrast, okadaic acid treatment in *msq* led to significantly increased ventricular FS of 20.8 ± 19.9% at 96 hpf (*p* = 0.01; Figure 5A).

Next, we performed immunoblotting assays in *msq* mutants after okadaic acid treatment to determine the levels of PKB phosphorylation at 96 hpf (Figure 5B). Similar to calyculin A treatment, PKB phosphorylation signal intensity was markedly higher in *msq* treated with okadaic acid compared to *msq* DMSO controls. Moreover, pPKB signal in okadaic acid treated *msq* embryos was comparable to their wild-type controls, indicating that this compound is, similar to calyculin A, a strong inhibitor of PKB dephosphorylation and therefore okadaic acid treatment preserved PKB phosphorylation in *msq* mutants in vivo.

### 3.3. Cyclosporine A is a Poor Modulator of Ventricular Fractional Shortening and PKB Phosphorylation in msq Mutants

In contrast to the compounds calyculin A and okadaic acid, which are known inhibitors of the protein phosphatases PP1 and PP2A, cyclosporine A is considered to interfere only with PP2A, implying that the impact of this compound on ventricular FS and PKB phosphorylation status in *msq* might be different to the before-analyzed compounds. According to the secondary screen for calyculin A and okadaic acid, we incubated wild-type zebrafish as well as *msq* mutants with cyclosporine A from 4 to 96 hpf. For wild-type zebrafish treated with DMSO only and wild-type zebrafish treated with cyclosporine A, we found no significant difference in ventricular FS (wt-DMSO: 48.5 ± 4.4% vs. wt-cyclosporine A: 48.2 ± 6.0%; *p* = 0.911; Figure 5C). Remarkably, ventricular FS tends to be higher in *msq* mutants after cyclosporine A treatment from 4 to 96 hpf (16.9 ± 16.2%, Figure 5C) compared to *msq* controls (7.9 ± 8.7%; *p* = 0.137). However, level of statistical significance with *p* < 0.05 was not reached in this experimental setting. In accordance, reconstitution of PKB phosphorylation in *msq* mutants was less pronounced after cyclosporine A administration than in *msq* embryos treated with okadaic acid or calyculin A (Figure 5D), indicating that cyclosporine A is a poor inhibitor of PKB dephosphorylation and cardiac contractility in *msq* mutants.

## 4. Discussion

Integrin-linked kinase is a key molecule of the mechanical stretch sensor in the vertebrate heart, regulating expression of stretch-responsive genes such as *anf* and *vegf* and thereby allowing adaption of cardiac contractility to various hemodynamic demands. As shown in several genetic studies in animal models as well as in humans, mutations in genes encoding for proteins of the mechanical stretch sensor system lead to reduced ventricular FS and finally to dilated cardiomyopathy [2,6,8,10].

Based on a mutation within the kinase domain of ILK homozygous mutant *msq* embryos display a progressive reduction of myocardial contractility. On molecular level, homozygous *msq* embryos are characterized by a reduced PKB phosphorylation and a decreased expression of the stretch responsive genes *anf* and *vegf* [10,15], making *msq* a suitable animal model for ILK-dependent DCM. Despite detailed characterization of the ILK-PKB signaling pathway, pharmacological approaches to restore ILK-PKB function are still missing.

With the aim to enhance ILK-PKB signaling pathway in *msq*, we studied the impact of 32 small compounds derived from a phosphatase inhibitor library on ventricular FS in this heart failure model. We hypothesized that the inhibition of dephosphorylation and consecutive inactivation of PKB by phosphatase inhibitors might lead to restored ILK-PKB signaling and finally to reconstituted cardiac contractility.

In our primary small compound screen using our recently established screening platform [21], we evaluated 32 compounds and found three biomolecules, namely calyculin A, okadaic acid and cyclosporine A significantly improving ventricular FS. Interestingly, these compounds are known to act on the protein phosphatases PP1 and PP2A, which are essential regulators of the phosphorylation status in numerous key signaling pathways [30,31,32]. Drug screening of other PP1/PP2A inhibitors such as the compounds B1 (cantharidic acid) and B2 (cantharidin) led to lethality of both wild-type and *msq* mutant embryos. We conclude that our straightforward small compound screening approach allows to identify appropriate potential therapeutic biomolecules and to exclude substances with adverse effects from further in vivo evaluation.

As observed in our secondary drug screening, we found that among the three identified compounds calyculin A turned out to have the strongest effect on cardiac contractility and PKB phosphorylation. However, in contrast to okadaic acid, it has been shown that calyculin A inhibits not only PP1 and PP2A but also the myosin light chain (MLC) phosphatase, which is an important regulator of the contractile apparatus in the vertebrate heart [30]. Myosin light chain phosphatase dephosphorylates the regulatory light chain of myosin II and initiates the relaxation process of muscle cells. Whether the inhibition of MLC phosphatase by calyculin A also contributes to restoration of ventricular FS in *msq* mutants was not analyzed in our experimental setting. However, we conclude that, due to its pleiotropic effects in cardiomyocytes, calyculin A should be considered as a promising biomolecule with the potential to treat ILK-dependent DCM.

Cyclosporine A is an effective immunosuppressive drug that has been prescribed for decades for a vast number of patients, e.g., after organ transplantation to reduce graft-versus-host reactions.

In contrast to calyculin A and okadaic acid, cyclosporine A failed to significantly increase ventricular FS in *msq* mutants despite recovered PKB phosphorylation. Hence, in our experimental setting cyclosporine A occurred only as an intermediate mediator of the ILK-PKB signaling pathway in comparison to calyculin A and okadaic acid. However, it is known that cyclosporine A has a beneficial myocardial effect by attenuating detrimental hypertrophy of the left ventricle in mice undergoing pressure overload [33]. Hence, based on the protective effect in cardiomyopathy model organisms and the long-term experience with cyclosporine A in daily clinical routine, cyclosporine A is still an interesting target for future investigations in the context of cardiomyopathies.

In recent years, the zebrafish has emerged as a powerful and reliable model organism for the rapid and straightforward in vivo analysis of small molecule bioactivity for a broad range of cardiovascular diseases. Advances in the field like fully-automated high-throughput screenings will be of additional advantage, enabling testing of numerous biomolecules in an effective and time-saving manner [23,34]. Although we show promising results of at least two compounds for the treatment of genetic ILK-dependent cardiomyopathy in a vertebrate model, additional studies in alternative model systems are needed to elucidate the transferability of our results to mammals and humans.

In this context, human-induced-pluripotent-stem-cell-derived cardiomyocytes cells (hiPSC-CM) have been successfully established in recent years for cardiovascular disease modeling as well as drug screening. In contrast to animal models, hiPSC-CM are biologically identical to their human donors, facilitating significantly the transferability of novel genetic and molecular findings. However, hiPSC-CM differ in several important aspects from adult human cardiomyocytes, especially in terms of maturation, gene expression or ion channel function, reducing their field of application [35]. In contrast, large mammalian animal models for cardiomyopathies, such as dogs, render pathomechanistical findings that are easy to transfer to humans based on the high interspecies homogeneities, but large-scale drug screening in a cost and time saving manner is not applicable in this type of model organism. Thus, we conclude that the different disease models, including cell-based approaches as well as vertebrate and mammalian animal models, with their particular strengths and weaknesses, should be seen as complementary in long-term drug discovery rather than exclusionary.

### Limitations

Initial drug screening with 32 compounds was partly performed with an arbitrarily predefined biomolecule concentration of 10 µM. This one-concentration-fits-all approach facilitates evaluating numerous compounds in a short time. However, biological impact of compounds that were lethal in our animal model might be overestimated and biological impact of compounds with no obvious effect on ventricular FS might be underestimated in our experimental setting due to inappropriate compound concentration.

## 5. Conclusions

Heart failure is one of the most frequent reasons for morbidity and mortality in developed countries. Genetic variants in the ILK gene are associated with impaired cardiac contractility in humans. Aided by the ILK-deficient zebrafish heart failure mutant (*msq*), we identified in an automated small compound screen the protein phosphatase inhibitors calyculin A and okadaic acid leading to significantly restored pump function of the zebrafish heart via reconstituting PKB phosphorylation. To evaluate the therapeutic potential of these promising small compounds in humans, calyculin A and okadaic acid should be further investigated in mammalian model organisms.

## Appendix A

**Table A1:** Modified ENZO ^®^ library of protein phosphatases inhibitors.

Plate Location	Name	Target	Lethality
A1	Calyculin A	PP1 and PP2A	
A2	Cyclosporin A	PP2A	
A3	Okaid acid	PP1 and PP2A	
B1	Cantharidic acid	PP1 and PP2A	wt, *msq*
B2	Cantharidin	PP1 and PP2A	wt, *msq*
B3	Endothall	PP2A	
B4	Benzylphosphonic acid	Tyrosine phosphatases	
B5	L-*p*-Bromotetramisole oxalate	Tyrosine phosphatases	
B6	RK-682	Tyrosine phosphatases	*msq*
B7	RWJ-60475	CD45 tyrosine phosphatase	
B8	RWJ-60475 (AM)3	CD45 tyrosine phosphatase (cell permeable)	
B9	Levamisole HCl	Mammalian alkaline phosphatase	
B10	Tetramisole HCl	Mammalian alkaline phosphatase	
B11	Cypermethrin	Calcineurin (PP2B)	
B12	Deltamethrin	Calcineurin (PP2B)	wt
C1	Fenvalerate	Calcineurin (PP2B)	
C2	Tyrphostin 8	Calcineurin (PP2B)	
C5	BN-82002		
C6	Shikonin		
C7	NSC-663284	CDC25	
C9	Pentamidine	PRL1	
C10	BVT-948	Tyrosine phosphatases	
C11	1-(2-Bromobenzyloxy)-4-bromo-2-benzylidene rhodanine	PRL3	
C12	Alexidine·2HCl	PTPMT1	wt, *msq*
D1	9,10-Phenanthrenequinone	CD45 tyrosine phosphatase	wt, *msq*
D2	BML-260	JSP-1	wt, *msq*
D3	Sanguinarine chloride	PP2C	wt, *msq*
D4	BML-267	PTP1B	
D5	BML-267 Ester	PTP1B (cell permeable)	
D6	OBA	Tyrosine phosphatases	
D7	OBA Ester	Tyrosine phosphatases (cell permeable)	
D8	Gossypol	Calcineurin (PP2B)	wt, *msq*
